# NTHL1 defines novel cancer syndrome

**DOI:** 10.18632/oncotarget.5864

**Published:** 2015-09-28

**Authors:** Roland P. Kuiper, Nicoline Hoogerbrugge

**Affiliations:** Department of Human Genetics, Radboud Institute for Molecular Life Sciences, Radboud university medical center, Nijmegen, The Netherlands

**Keywords:** Chromosome Section, base excision repair, polyposis, NTHL1

A small minority of the population is at a highly increased risk of developing colorectal cancer (CRC) due to the presence of genetic risk factors [1, 2]. These patients usually present with cancer at earlier age compared to the normal population, multiple tumors during their lives, and/ or a positive history of cancer in the family. A subset of the patients also presents with increased numbers of polyps in the colon or rectum, referred to as polyposis. Currently, approximately 5% of CRCs can be explained by germline mutations in one of the high-penetrance CRC predisposing genes. The identification of such mutations, and thereby the diagnosis of a hereditary CRC syndrome, greatly facilitates the clinical management of patients and their relatives, leading to better risk estimates for the cancers that are involved, and early detection or even prevention of cancer. Unfortunately, however, a substantial proportion of the suspected patients and families remains unexplained and many efforts are taken to uncover their genetic origin.

We recently performed exome sequencing on 51 highly suspected patients diagnosed with polyposis, a strong indication for a genetic predisposition. Selection criteria were further constricted by including only patients with a specific morphological subtype of polyps, i.e., conventional adenomas, particularly since known polyposis-associated syndromes have shown specificity for polyp type. We identified a homozygous nonsense mutation (c.268C>T encoding p.Q90*) in the base excision repair gene *NTHL1* in three unrelated families [3]. The high penetrance of this mutation was demonstrated by a complete cosegregation with the phenotype: all seven homozygotes had adenomatous polyps, six had (multiple) malignancies including CRC, and none of the heterozygous or wild-type family members were affected. Furthermore, in concordance with a base excision repair defect, both adenomas and CRCs from these patients showed a specific mutation spectrum in CRC driver genes: an almost complete bias towards C>T transitions. Therefore, biallelic germline truncating mutations in *NTHL1* underlie a novel recessive adenomatous polyposis and CRC predisposition syndrome [3], which we refer to as *NTHL1*-associated polyposis (NAP).

Based on the prevalence of the pQ90* variant in the Dutch population, we estimated that homozygosity would occur once in every ~75,000 individuals, representing >200 affected individuals [3]. Other potentially pathogenic *NTHL1* variants appear to be less prevalent, suggesting that this mutation is the main contributor to NAP in the Netherlands. In other populations, particularly those that are less well represented in exome sequencing studies reported so far, this may be different.

DNA repair defects appear to be a common theme in CRC syndromes [1, 2]. Most prevalent are mutations in the mismatch repair genes causing Lynch syndrome (LS), which predisposes to CRC in the absence of polyposis. Biallelic mutations in the base excision repair gene *MUTYH* are the cause of *MUTYH*-associated polyposis (MAP), and germline missense mutations in the proofreading domains of the *POLE* and *POLD1* genes were recently shown to cause polymerase proofreading-associated polyposis (PPAP) [4]. Besides CRC, these syndromes also show increased incidences of extracolonic cancers, which is particularly true for LS, where cancers of the endometrium, small bowel, stomach, ovary, upper urological tract and several others are frequently observed. Furthermore, PPAP appears to be associated with an increased incidence of endometrial cancers [2, 4], and for MAP malignancies of the duodenum, ovary, bladder, and skin have been described, occasionally even resembling the phenotype of LS [5]. Our preliminary data on the three families with NAP also point towards an extended spectrum of cancers, which may include malignancies of the endometrium, duodenum, skin (basal cell carcinoma), and several others. Obviously, these findings warrant further investigation once more NAP families have been identified. These studies should also reveal whether the presence of conventional adenomas, an inclusion criterion in our study, indeed is typical for NAP.

The strongly biased mutation spectrum observed in adenomas and carcinomas not only provides insight into the mechanism underlying tumor risk, but also provides a useful strategy to establish whether (extracolonic) tumors observed in NAP patients are indeed caused by a NTHL1 defect. Furthermore, the detection of a mutation bias may assist in the future identification of novel patients and families with base excision repair defects, and the discrimination between NAP (C>T transitions) and MAP (G>T transversions). The observation of opposite mutation spectra between NAP and MAP is striking, and may be explained by differences in substrate specificity of the two DNA glycosylases [3, 6, 7]. MUTYH is specifically required for recognition and correction of mismatched adenine bases opposite oxidation-damaged guanine bases (8-oxoG), which explains why G>T transversions accumulate in MAP patients. NTHL1, in contrast, removes oxidized pyrimidines and ring-opened purines, and the activity towards 8-oxoG appears to be absent [7]. However, much remains to be learned about the role of NTHL1 deficiency in tumorigenesis within the colorectum and other tissues.

In conclusion, with the discovery of three adenomatous polyposis families with homozygous NTHL1 p.Q90* mutations we have identified a novel recessive polyposis/CRC syndrome, which appears to be associated with a broad (LS-like) tumor spectrum. Future research will undoubtedly reveal more about the prevalence of NAP, its cancer risk, and its tumor spectrum. Considering the recessive inheritance and the broad tumor spectrum, one might consider NAP also in the absence of a positive family history, and in cases with (multiple/early-onset) cancers other than CRC.
